# Supplementary material for: Seed quality drives grain yield in Ethiopian and Senegalese sorghum: Insights from machine learning
Source: PLoS One. 2025 Aug 14;20(8):e0329366. doi: 10.1371/journal.pone.0329366 (PMC12352784; doi:10.1371/journal.pone.0329366)
Supplement: Attachment — Submitted filename: Plos one R3 response.pdf [file pone.0329366.s006.pdf]

Dear Dr. Somashekhar Mallikarjun Punnuri,

Thank you for the opportunity to revise our manuscript, "Seed quality drives grain yield in Ethiopian and Senegalese sorghum: Insights from machine learning" (PONE-D-24-50599R2). We are grateful to all the reviewers for their time and valuable feedback.

We would like to extend our special gratitude to Reviewer #4 for their exceptionally thorough and insightful review. Their detailed suggestions have been invaluable and have significantly improved the clarity, rigor, and overall quality of our manuscript. We have carefully addressed all comments and believe the revised manuscript is now much stronger. Below, you will find our point-by-point responses to the comments from Reviewer #4.

Reviewer #4: For Title and Author Details:

Capitalized each word in the title per academic style.
Answer: We appreciate the suggestion. We have double-checked the title against the PLOS ONE formatting guidelines and believe the current sentence-case formatting is consistent with the journal's style.

Corrected spacing in names ("Adama R Tukuli" → "Adama R. Tukuli").
Answer: Thank you. This has been corrected in the revised manuscript.

Added a missing comma before the last author.
Answer: Thank you for noting this. We believe this comment may refer to a previous version of the manuscript, as the comma is present in the current version. We have confirmed the formatting is now correct.

Ensured consistent superscripts (^{1}, ^{2}, etc.) and symbols (†, *) for affiliations and contributions.
Answer: Thank you. We have reviewed all author affiliations and confirmed their consistency.

Introduction:
(with highlighted suggestions)

Sorghum (Sorghum bicolor (L.) Moench) is a vital food and fodder source in Africa and Asia. Globally, it's more common as animal feed but is gaining attention as a biofuel crop [1].
Answer: Thank you for your comment. We revised the sentence as suggested.

With over 60 million tonnes produced annually and Africa contributing ~20 million tonnes, it ranks second only to maize on the continent [2].
Answer: Thank you for your comment. We revised the sentence as suggested.

Sorghum is vulnerable to fungal diseases that reduce yield and quality [3].
Answer: Thank you for your comment. We revised the sentence as suggested.
Key diseases include anthracnose, grain mold, and rust. Colletotrichum sublineola, causing anthracnose, can reduce yields by up to 70% in hot, humid conditions [4].
Answer: Thank you for your comment. We revised the sentence as suggested.

(Clarify sentence structure for better flow.) It spreads easily due to its resilience and dispersal by wind/water [4].
Answer: Thank you for your comment. We revised the sentence as suggested.

Fusarium spp., a common grain mold pathogen, produces mycotoxins like fumonisins—a food safety risk in regions highly dependent on sorghum [5].
Answer: Thank you for your comment. We revised the sentence as suggested.

Losses range from 30% to 100% depending on multiple factors [6,7]. (Replace "staggering" with neutral term.)
Answer: Thank you for your comment. We revised the sentence as suggested.

Rust (Puccinia purpurea) appears as rust-like spots and causes yield loss up to 65%, depending on plant maturity and conditions [6,7].
Answer: Thank you for your comment. We revised the sentence as suggested.

(Clarify "unfavorably mature" wording.)
Germplasm-based resistance is seen as the most effective control strategy [7].
Answer: Thank you for your comment. We revised the sentence as suggested.

Resistant genotypes have been identified through screening and breeding efforts [6].
Answer: Thank you for your comment. We revised the sentence as suggested.

Our previous study evaluated 179 accessions from Ethiopia, Gambia, and Senegal. Traits included yield, seed weight, flowering time, germination, panicle traits, and disease resistance [8,9].
Answer: Thank you for your comment. We revised the sentence as suggested.

Due to limitations in basic statistical analysis, we applied machine learning to explore complex relationships and identify the best algorithm for trait evaluation.
Answer: Thank you for your comment. We revised the sentence as suggested.

The Materials & Methods Section:

It provides a comprehensive description of the data and analytical approach. The data were collected from field trials in Isabela, Puerto Rico, involving 179 sorghum accessions from

Ethiopia, Gambia, and Senegal, with various phenotypic traits measured. However, the mention of “including controls 201 accessions” is unclear and should be rephrased or separated for clarity to specify the total sample size and the role of controls. The description of missing data handling using Multivariate Normal Imputation is appropriate, but it would be beneficial to briefly justify the choice of this method and mention any criteria used for missing data filtering. Disease resistance scoring on a 1-5 scale is noted, but it would strengthen the methodology to indicate whether this scale is a standard or validated measure in sorghum phenotyping.
Answer: We thank the reviewer for these valuable suggestions. The text has been rephrased to clearly state the total sample size (201), which includes the 179 primary accessions and the 22 control lines used for comparison. Also, we have added a justification for using Multivariate Normal Imputation, explaining that it was chosen to preserve the covariance structure among the phenotypic traits. We have now specified that the 1-5 disease resistance scale follows standard, validated protocols detailed in our previous publications

The statistical analysis section mentions t-tests comparing accessions from Ethiopia and Senegal, but the rationale for excluding Gambia accessions from this comparison should be explained. The use of PCA and hierarchical clustering is suitable for exploring trait relationships, though the method for selecting the number of clusters (four clusters) should be clarified, for example, by referencing dendrogram inspection or cluster validity indices.
Answer: We thank the reviewer for pointing out these areas needing clarification. We have revised the "Statistical analysis" section to address both points. We have added a statement explaining that the Gambian accessions were excluded from the *t*-test comparison due to their small sample size (n=12). We have also clarified that the choice of four clusters was based on a visual inspection of the dendrogram, as this approach provided a logical balance between cluster separation and a manageable number of groups for our subsequent analyses.

The machine learning workflow is well-outlined and supported by Figure 1, although the figure caption is embedded in the text and should be moved to the figure legend area with formal formatting. In cluster classification, a pre-balanced dataset of 248 points was used with undersampling, but it is not clear how this number was derived, so explanation of the balancing process is recommended. The use of stratified splits and GridSearchCV for hyperparameter tuning is good practice, yet including the rationale for using macro F1-score as the evaluation metric would enhance transparency. The study applies several machine learning models spanning linear, kernel-based, ensemble, and instance-based methods, which is commendable for model comparison. However, it would be helpful to state whether default hyperparameters were used initially and consider summarizing model characteristics in a table for reader clarity.
Answer: We thank the reviewer for these excellent suggestions. We have now explicitly explained in the "Random undersampling" section how the 248-point dataset was derived (by balancing all four clusters to the size of the smallest cluster, n= 62). We also have added a justification for using the macro F1-score, noting its robustness for multi-class classification with potential class imbalance. We further clarified that default hyperparameters were used in the

initial screening process before tuning and, as suggested, have added a new Table 1 to summarize the characteristics of the ML models for enhanced reader clarity.

The Results section provides a thorough and well-organized presentation of the phenotypic diversity analyses using PCA, hierarchical clustering, and machine learning classification. The interpretation of the PCA results is clear, particularly in describing the variance explained by the first two principal components and the clustering of traits. However, it would be beneficial to briefly discuss the implications of the remaining unexplained variance and whether further principal components were considered or excluded, to give a fuller picture of the data structure. Additionally, while the PCA biplot explanation outlines trait clusters, clarifying the direction and nature of trait correlations with the principal components would help readers interpret the loading plots more effectively.
Answer: We thank the reviewer for these insightful suggestions to improve the interpretation of our PCA results. We have added a sentence to the Results section acknowledging that while PC1 and PC2 explain 47.6% of the variance, other components capture additional variation. We clarify that our focus on the first two components is for effectively visualizing the primary patterns in the data. We have also expanded our description of the loading plot (Fig. 2b) to explicitly explain how the direction and length of the trait vectors relate to their correlation with the principal components, using specific examples from the plot to guide the reader.

The comparative analysis between Ethiopian and Senegalese accessions is well-supported by statistical evidence, but the presentation of Table 1 could be improved for better readability. Restructuring the table, possibly by separating phenotypic and genotypic traits or enhancing column headers, would make it easier for readers to digest the information. Furthermore, although the statistical differences are clear, adding brief commentary on the biological or agronomic significance of these differences would strengthen the relevance of the findings.
Answer: We thank the reviewer for this excellent feedback to improve the presentation and impact of our results. As suggested, Table 1 has been completely restructured into a vertical format with grouped traits to enhance readability. A new paragraph has been added immediately following the table to discuss the potential biological and agronomic significance of the observed phenotypic differences between the two regions.

The hierarchical clustering analysis is appropriately detailed, and the use of the DIANA algorithm is well explained. Regarding the machine learning classification, the section clearly justifies the need for a two-step approach due to initial low accuracy. However, mentioning which algorithms were initially tested and elaborating on how class imbalance and the feature-to-sample ratio affected performance would provide better context and insight into the challenges encountered. Finally, when referring to figures throughout the section, ensuring detailed and accessible figure legends close to the figures will facilitate cross-referencing and enhance reader understanding.
Answer: We thank the reviewer for these suggestions. We have revised the "Machine learning-based classification" section to specify the algorithms that were initially tested in our

preliminary analysis. We have also elaborated on how the severe class imbalance (with each accession as a unique class) and the feature-to-sample ratio led to the low accuracy in this initial step, justifying our two-step approach. Furthermore, we have reviewed all figure legends to ensure they are detailed and correctly placed with their corresponding figures to improve readability.

The discussion effectively highlights the importance of sorghum for food security in dry tropics, particularly Ethiopia, and appropriately connects previous findings on yield declines due to biotic and abiotic stresses. The explanation of geographic patterns in phenotypic diversity using PCA and clustering is well presented and shows thoughtful interpretation. However, the section would benefit from more clearly separating speculative interpretations from established results to avoid potential overstatements. For example, while cluster interpretations are insightful, explicitly stating that these are hypotheses requiring further validation, ideally with genotypic data, would strengthen the discussion. The references to related machine learning studies demonstrate good awareness of the field, but the comparison between prior studies and the current more granular classification attempt could be expanded to clarify why phenotypic trait overlap limited classification accuracy here.
Answer: We thank the reviewer for this insightful feedback. We have added explicit statements to the paragraph interpreting the clusters to clarify that these are hypotheses requiring further validation with genotypic data, as suggested. We have also expanded the discussion to better explain why our initial, more granular classification attempt had low accuracy, clarifying that the significant phenotypic overlap among the 179 diverse accessions made this a much more difficult task than classifying a small number of distinct hybrids.

The machine learning approach is described in detail, with a good rationale for model selection. Yet, the discussion could improve by including more critical reflection on limitations, such as the relatively small sample size per class and the low number of traits, which constrained classification performance. Additionally, the transition from unsuccessful fine-grained classification to clustering and then classification of clusters is logical but would be clearer if the rationale for choosing four clusters was explicitly justified. The mention of model performances is useful, though the discussion appears to be cut off abruptly; completing the results summary and relating model outcomes back to biological relevance or breeding applications would enhance the narrative. Finally, better signposting within the discussion through subheadings or paragraph breaks would improve readability, given the density of information.
Answer: We thank the reviewer for their valuable suggestions to improve the Discussion section. In the revised manuscript, we have restructured the entire section with new subheadings for clarity, including a dedicated 'Limitations and Future Directions' section for critical reflection. We have also expanded on our classification results to discuss their biological relevance and applications in breeding. The rationale for selecting four clusters has been clarified in the Materials and Methods section, as requested in a previous point.

The conclusion effectively summarizes the study’s key findings and emphasizes the potential of

machine learning to enhance sorghum breeding through improved germplasm characterization and yield prediction. The recognition of the Neural Boosted model's superior performance and the identification of seed weight and germination rate as critical yield determinants are well highlighted. However, the conclusion could be strengthened by explicitly acknowledging limitations, such as the study's focus on only Ethiopian and Senegalese accessions and the relatively small phenotypic trait set, which may affect the generalizability of the findings. Additionally, while the broader applicability to other cereal crops is mentioned, providing specific examples or cautionary notes about differences in crop biology would make this claim more balanced.
Answer: We thank the reviewer for their valuable suggestions to make our Conclusion more balanced and comprehensive. In the revised manuscript, we have now explicitly acknowledged the study's limitations regarding generalizability and have added a cautionary note about the broader applicability of our findings to other cereal crops, considering potential differences in crop biology as suggested.

The discussion of disease resistance traits' limited influence is insightful, but further elaboration on possible reasons—such as environmental conditions or disease prevalence during trials—would clarify these findings. The application of ADASYN to address class imbalance is a notable methodological strength; however, the conclusion would benefit from a brief mention of potential challenges or biases introduced by synthetic data generation. The practical recommendations about prioritizing seed quality and establishing local seed testing are valuable and grounded in the results, yet highlighting potential implementation challenges in resource-limited settings could add nuance.
Answer: We thank the reviewer for their insightful suggestions to add more nuance to our Conclusion. In the revised manuscript, we have elaborated on the potential reasons for the limited influence of disease resistance traits, included a cautionary note on the potential biases of ADASYN, and acknowledged the implementation challenges for our practical recommendations in resource-limited settings.

Finally, the call for future integration of multi-modal data is well placed but could be enhanced by suggesting concrete next steps or specific types of data integration that would most benefit sorghum breeding. Overall, the conclusion provides a solid wrap-up but would improve with clearer acknowledgment of limitations and a more nuanced discussion of the broader implications and future directions.
Answer: We thank the reviewer for this excellent suggestion to make our future directions more concrete. As recommended, we have revised the final sentences of our Conclusion to suggest specific types of data for integration (GBS, environmental variables) and concrete analytical next steps (GWAS, genomic selection models) that would most benefit future sorghum breeding efforts.
