# Supplementary material for: Seed quality drives grain yield in Ethiopian and Senegalese sorghum: Insights from machine learning
Source: PLoS One. 2025 Aug 14;20(8):e0329366. doi: 10.1371/journal.pone.0329366 (PMC12352784; doi:10.1371/journal.pone.0329366)
Supplement: Attachment — Submitted filename: Review.docx [file pone.0329366.s001.docx]

First of all, I congratulate all the authors on their excellent work. Some corrections are required for a better presentation of the work. The recommendations are given below:

1. Modify the abstract and include some key findings in this section.
2. Update the introduction with recent work and references.
3. The discussion section requires current-year references.
4. Separate the conclusion section and modify it with specific recommendations for sustainable agricultural practices in the study area.
5. Figure 3 is not visible; update it with a higher resolution for better understanding.
6. In Figure 4, the x and y axes need to be clearer (explain what "Predicted" and "Actual" mean, including their units or variables).
7. Clearly mention all data sources specifically in data source section.
8. Mention the level of significance of the p-value below Table 1.
9. Describe the selection procedure for the 179 observations.
