# Supplementary material for: Seed quality drives grain yield in Ethiopian and Senegalese sorghum: Insights from machine learning
Source: PLoS One. 2025 Aug 14;20(8):e0329366. doi: 10.1371/journal.pone.0329366 (PMC12352784; doi:10.1371/journal.pone.0329366)
Supplement: Attachment — Submitted filename: Prom2017 revisit response to reviewer.docx [file pone.0329366.s004.docx]

**Reviewer #1:** Ensure proper use of acronyms in the text

e.g. if Boosted Tree (XGBoost) then XGBoost can be used consistently in the text.

Methodology can be improved

1. The significance of R-squared and RMSE for assessing clustering i.e. classification results is not sufficiently explained

**A: We thank the reviewer for their careful attention to detail. We have reviewed the manuscript and ensured consistent use of acronyms throughout the text.**

2. Citations for JMP 17 Pro, if available would be helpful for readers

Results section can be improved

1. Table 1. Respective units for each treats can be given for better understanding.

**A: Thank you so much for pointing it out. I've added the units to Table 1 for clarity.**

2. Large difference in accuracy of grain yield prediction models is not sufficiently explained.

**A: We thank the reviewer for this suggestion. We have added a citation for JMP Pro 17 to the Materials and Methods section**

**Reviewer #2:** First of all, I congratulate all the authors on their excellent work. Some corrections are required for a better presentation of the work. The recommendations are given below:

1. Modify the abstract and include some key findings in this section.

**A: We thank the reviewer for this suggestion. We have revised the abstract to include more specific key findings, including the identification of a Neural Boosted model as the best performer for yield prediction and its associated, cross-validated R-squared and RMSE values. The abstract now more accurately reflects the most important results of our study.**

2. Update the introduction with recent work and references. & 3. The discussion section requires current-year references.

**A: We thank the reviewer for highlighting the need to update our references. We have thoroughly reviewed the Introduction and Discussion sections and incorporated relevant literature published in recent years**

4. Separate the conclusion section and modify it with specific recommendations for sustainable agricultural practices in the study area.

**A: We thank the reviewer for their insightful feedback. We have revised the conclusion.**

5. Figure 3 is not visible; update it with a higher resolution for better understanding.

**A: I will make sure to upload the original image with the highest resolution for the final version.**

6. In Figure 4, the x and y axes need to be clearer (explain what "Predicted" and "Actual" mean, including their units or variables).

**A: We thank the reviewer for their comment regarding Figure 4. During the revision process, and in response to other reviewer feedback, Figure 4 has been removed from the manuscript.**

7. Clearly mention all data sources specifically in data source section.

**A: We thank the reviewer for this suggestion. We have clarified in the Materials and Methods section that the phenotypic data used in this study, originally collected and analyzed in our previous publications**

8. Mention the level of significance of the p-value below Table 1.

**A: We thank the reviewer for this suggestion. We have added a statement to the caption of Table 1 indicating that the level of significance for the *p*-values is p < 0.05.**

9. Describe the selection procedure for the 179 observations.

**A: We thank the reviewer for requesting clarification on the selection of the sorghum accessions. The 179 accessions used in this study were obtained from the USDA-ARS Plant Genetic Resources Conservation Unit in Griffin, Georgia, where they are maintained as part of a larger sorghum collection. These accessions, originating from Ethiopia, Gambia, and Senegal, were selected for evaluation based on traits including grain yield, seed weight, plant height, panicle length, and flowering time, considered as priority traits for the National Plant Germplasm System sorghum collection, and our previous study included evaluation of their disease resistance. We added a more detailed description in materials and methods.**

**Reviewer #3:** The manuscript titled explores the application of various machine learning models, including Random Forest, Boosted Tree, and DBSCAN, to cluster 179 sorghum accessions based on phenotypic traits and predict grain yield. The study identifies distinct clustering patterns and highlights seed weight and germination rate as key predictors of yield, with the Boosted Tree model demonstrating superior performance. While the research showcases the potential of ML in sorghum germplasm characterization, issues related to methodological justification, data handling, model validation, and biological interpretation limit its current impact. The manuscript requires major revision to address these critical concerns. First of all, I have few questions.

• How do the identified clusters translate into meaningful biological or agronomic categories? Are they linked to specific landraces, environmental conditions, or genetic traits?

**A: The identified clusters provide insights into the genetic diversity and potential agronomic value of the sorghum accessions. Our analysis considered both phenotypic traits and country of origin, allowing us to explore the relationships between these factors. While Figure 1a (PCA plot) shows a considerable overlap between Ethiopian and Senegalese accessions, Table 1 reveals statistically significant differences in various traits between genotypes from these two countries. This suggests that despite some phenotypic similarities, there is an underlying genetic variation between the two groups.**

**The cluster analysis, which integrated information from all traits, further supports this notion. By considering the overall phenotypic profiles, the clustering algorithm could differentiate between accessions from Ethiopia and Senegal, even though they might appear similar based on a subset of traits visualized in the PCA plot. This highlights the importance of considering multiple traits simultaneously when assessing genetic diversity and agronomic potential.**

• Why were random undersampling and basic imputation methods chosen over more advanced techniques that preserve data integrity and variability?

**A: We thank you for the reviewer’s comment. We addressed the observed class imbalance, where Groups 1 and 4 significantly outnumbered Groups 2 and 3, using the ADASYN technique.**

• How do the authors explain the exceptionally high R² value (>0.90) in yield prediction? Could this indicate overfitting, and how was this risk mitigated?

**A: The exceptionally high R² value (>0.90) observed initially in the Boosted Tree model for yield prediction indeed raised concerns about potential overfitting. This could be attributed to the model's complexity and its tendency to capture noise in the training data, leading to inflated performance estimates. To mitigate this risk, we re-evaluated the model using k-fold cross-validation with k=5 and 10 repeats. This technique provides a more robust assessment of the model's performance on unseen data by repeatedly training and testing it on different subsets of the data. The cross-validation results showed a lower average R² of approximately 0.48, which is a more realistic estimate of the model's predictive accuracy. This indicates that the initial high R² might have been partly due to overfitting, and the cross-validation approach helped to provide a more reliable evaluation.**

• Were external validation datasets considered to confirm the generalizability of the clustering and prediction models? If not, why?

**A: While external validation datasets would be ideal for confirming the generalizability of our clustering and prediction models, we unfortunately lack access to such datasets with directly comparable traits and environmental conditions. However, we acknowledge the importance of external validation and plan to explore potential sources of comparable data in future research.**

• How do the findings align or contrast with existing literature on sorghum yield prediction and clustering? Are there unexpected results that warrant further investigation?

**A: We thank the reviewer for this insightful question. Our findings on seed weight and germination rate as key yield predictors align with existing literature, as does the success of the Boosted Tree model; however, the lower importance of disease resistance in our yield model contrasts with some studies, potentially due to lower disease pressure in our trials or the model's prioritization of traits. The variable clustering performance across algorithms, particularly the lower-than-expected performance of SVMs and neural networks, and the generally low Silhouette scores, highlight the complexity of sorghum germplasm diversity and warrant further investigation, as detailed in the revised discussion.**

• Add overall methodological flowchart for better understanding to the readers

**A: We appreciate the reviewer's suggestion regarding a methodological flowchart. We have added a flowchart (Figure 1) to visually represent the main stages of our analysis.**

• While the introduction outlines the importance of sorghum and the application of machine learning (ML), it does not critically engage with the why behind the use of ML in this specific context. What inherent limitations of traditional phenotypic analysis methods motivated this study? For instance, are traditional clustering techniques insufficient due to non-linearity or high-dimensional trait interactions. This needs to be explicitly articulated to establish a stronger foundation for the study’s relevance.

**A: We thank the reviewer for raising this important point about the motivation for using machine learning. We have revised the Introduction to explicitly state the limitations of traditional phenotypic analysis methods that prompted this study. Specifically, traditional approaches can be labor-intensive, time-consuming, and may struggle to capture complex, non-linear relationships and high-dimensional trait interactions, which machine learning methods are better equipped to handle. This clarification strengthens the rationale for our approach, as now presented in the revised Introduction.**

• The introduction lacks clearly defined research questions or hypotheses. What specific biological or agronomic insights were the authors expecting to uncover through clustering and yield prediction? A clearly articulated hypothesis would help guide the reader through the manuscript's logical flow.

**A: We appreciate the reviewer's suggestion to clarify our research objectives. We have revised the Introduction to include explicit research questions that guided our study.**

• There’s little discussion on how ML has been applied in other crop-related studies, particularly in African germplasm contexts. Are there comparable studies in maize, millet, or other cereals? Adding such comparative insights could strengthen the rationale for selecting sorghum and the specific ML techniques.

**A: We thank the reviewer for suggesting we broaden the context of our work. We have revised the Introduction to include a discussion of machine learning applications in other crop-related studies, particularly those focusing on African germplasm of maize, pearl millet, and rice.**

• The manuscript applies a wide range of ML models (Random Forest, DBSCAN, Boosted Trees, etc.) without adequately explaining the selection criteria. Why were these models chosen over others, such as Gradient Boosting Machines, LightGBM, or even deep learning approaches like CNNs, which have shown promise in similar agricultural datasets?

**A: The choice of models (Random Forest, Boosted Trees, SVM) was guided by their strengths in revealing feature importance, predictive accuracy, and versatility for both classification and regression tasks, respectively.**

**Gradient Boosting Machines and LightGBM were not included as they were anticipated to yield similar results to the boosting methods already employed. CNNs are primarily designed for image data, which was not used in this study. DBSCAN was excluded due to its sensitivity to noise and the challenges of hyperparameter tuning.**

• The handling of missing data via Multivariate Normal Imputation is mentioned briefly. However, why was this specific imputation method chosen? Were other imputation strategies (e.g., k-NN imputation, MICE) considered, and how might the choice of imputation affect downstream clustering and prediction outcomes?

**A: Multivariate Normal Imputation was selected for its ability to preserve variable relationships and its computational efficiency. Our data suggests an approximate normal distribution for many variables, which aligns with MVNI's assumptions.**

• The study uses random undersampling to address class imbalance, which is problematic as it can discard potentially informative data. Did the authors consider alternative methods like SMOTE, ADASYN, or cost-sensitive algorithms that preserve data integrity? If not, why? Moreover, how does the undersampling affect model generalizability, especially given the small sample size?

**A: We appreciate the reviewer's comment on alternative class imbalance methods. We included random undersampling primarily as a computationally efficient baseline to compare against ADASYN, an oversampling technique that preserves all original data. This direct comparison allows us to assess the impact of potential information loss from undersampling.**

• While GridSearchCV is referenced, critical details are missing- What was the cross-validation strategy (e.g., k-fold, stratified k-fold)? Were metrics like precision, recall, F1-score considered in addition to RMSE and R²? Were there any issues with overfitting, especially given the high performance of the Boosted Tree model?

**A: We thank the reviewer for their insightful questions regarding our model evaluation. As detailed in the revised manuscript, we utilized *stratified* k-fold cross-validation: 5-fold for undersampling and nested 5-fold (outer) / 3-fold (inner) for ADASYN, ensuring representative class distributions. For clustering, we evaluated models using precision, recall, F1-score (macro-averaged), and accuracy, selecting the best model via GridSearchCV based on F1-score.**

• The manuscript heavily focuses on RMSE and R² for model evaluation. However, these metrics alone do not provide a complete picture. For clustering, did the authors consider internal validation metrics like the Silhouette Score, Davies-Bouldin Index, or Dunn Index to assess cluster quality?

**A: We acknowledge the reviewer's concern about relying solely on RMSE and R² for model evaluation. To address this, we have included additional metrics such as average R², average RMSE, standard deviation of RMSE, and highest R² across the k-fold cross-validation (k=5, repeats=10) for the grain yield prediction models. This provides a more comprehensive view of the model's performance and its consistency across different data subsets. Also, we have now included the Silhouette Score as an internal validation metric to assess the quality of our clustering results. This metric is calculated for each clustering method and reported in the manuscript.**

• The clustering results identify distinct groups, but what do these clusters mean biologically or agronomically? Are the clusters linked to known ecotypes, landrace origins, or environmental adaptations? Without such context, the clustering analysis feels descriptive rather than explanatory.

**A: We appreciate the reviewer's point regarding the explanatory power of our clustering analysis. We have added a statement to the Discussion section explicitly clarifying that the dendrogram analyses were primarily intended as an *exploratory* tool to visualize relationships and guide future research, rather than to provide definitive biological explanations (see revised manuscript). While the clustering identifies groups with similar phenotypic profiles, the goal was to generate hypotheses and explore alternative perspectives on germplasm organization. The discrepancies between clustering outcomes and supervised classification performance further highlight the complexity of trait relationships, informing model selection for future studies.**

• The Boosted Tree model achieved an exceptionally high R² (0.9026) for yield prediction. This raises concerns about potential overfitting, especially since real-world agricultural yield data is notoriously noisy. Was a learning curve analysis conducted to ensure model robustness? How does the model perform on truly unseen data (e.g., through external validation or time-based splits)?

**A: Thank you for pointing out the exceptionally high R² for the Boosted Tree model, which indeed raised concerns about potential overfitting. We acknowledge that the initial R² of 0.9026 was obtained on the training set, and we appreciate your suggestion to conduct a more robust evaluation.**

**To address this, we re-ran the analysis in JMP Pro 17 using k-fold cross-validation with k=5 and 10 repeats. This approach provides a more realistic estimate of the model's performance on unseen data. The cross-validation results showed a reduced R² of approximately 0.48 for the Boosted Tree, Neural Boosted, and Bootstrap Forest models. While this is lower than the initial estimate, it still indicates a reasonable predictive ability considering the inherent noise in agricultural yield data.**

• There’s no discussion of prediction errors. Were there specific accessions where the model consistently under- or over-predicted yield? Error analysis could reveal important insights into model biases or data limitations.

**A: Thank you for raising the important point about analyzing prediction errors. We agree that examining the variability in prediction accuracy can reveal valuable insights into model stability and potential biases.**

**To address this, we have now included the average R-squared, average RMSE, and the standard deviation of RMSE across the k-fold cross-validation (k=5, repeats=10) for the grain yield prediction models. This provides a more comprehensive view of the model's performance and its consistency across different data subsets. We have also added a discussion of the potential factors contributing to the observed variability in prediction errors, such as specific trait combinations or environmental influences.**

• The discussion is largely descriptive, summarizing results without linking them to broader agronomic or biological theories. For instance, why might seed weight and germination rate be the most important predictors of yield? Are these findings consistent with physiological models of plant growth or prior breeding studies?

**A: We thank the reviewer for highlighting the need to connect our findings to broader biological principles. As now discussed in the revised manuscript, the importance of seed weight and germination rate as yield predictors is consistent with established physiological understanding. Larger seeds typically contain greater nutrient reserves, fueling vigorous early growth, while a higher germination rate ensures a denser plant stand, both contributing to greater yield potential. We have added a paragraph to the Discussion section explicitly linking these traits to underlying physiological mechanisms and citing relevant literature.**

• The manuscript claims that ML models “highlight the potential for efficient germplasm characterization,” yet the models were not externally validated. Without independent validation, this conclusion is speculative. The discussion should temper such claims and acknowledge limitations.

**A: We appreciate the reviewer's comment regarding the need for external validation to support our claims about efficient germplasm characterization. While we employed k-fold cross-validation to assess model performance on unseen data, we acknowledge that this does not fully replace validation on a truly independent dataset. In the revised manuscript, we have tempered our claims and explicitly stated the limitations of the current validation approach. We recognize the importance of external validation for demonstrating the generalizability of our findings and plan to pursue this in future research.**

• The manuscript does not critically reflect on its own methodological limitations. Issues such as potential overfitting, the limitations of undersampling, or biases in phenotypic data collection are not discussed. A more self-reflective section acknowledging these limitations would enhance the manuscript’s credibility.

**A: We appreciate the reviewer's insightful comments regarding the need for a more critical reflection on methodological limitations. We acknowledge that the manuscript could benefit from a more in-depth discussion of potential biases and limitations associated with our approach. In response to this feedback, we have revised the Discussion section to include a more thorough analysis of the limitations of our study.**

• The manuscript lacks sufficient detail for reproducibility. For example- Were random seeds set during model training to ensure reproducibility? What software versions and libraries were used (e.g., specific versions of Scikit-learn, JMP Pro)? Can the code pipeline be shared to allow other researchers to replicate the analysis?

**A: We appreciate the reviewer's emphasis on reproducibility. As detailed in the revised Materials and Methods section, we have now explicitly specified the random seeds used throughout the analysis (e.g., random_state=42 for data splitting and model initialization) in both the undersampling and ADASYN implementations. The specific versions of key libraries used, including scikit-learn (version 1.2.2), imblearn (version 0.10.1), and JMP Pro (version 17), are also now included. While the initial submission did not include the code, we are happy to share the complete Python scripts and JMP Pro files upon request to facilitate full replication of our analysis.**

**Reviewer #4:** Review with Suggestions:

TITLE:

Machine Learning for Clustering and Yield Prediction in Ethiopian and Senegal Sorghum Collections

This study presents an in-depth application of machine learning (ML) for clustering and yield prediction in Ethiopian and Senegalese sorghum accessions. The research is well-structured, employing diverse ML models and statistical techniques to analyze phenotypic traits. However, there are areas where clarifications, additional justifications, and refinements would improve the study’s rigor and applicability.

TITLE, ABSTRACT & AUTHOR INFORMATION:

The Title is informative and accurately conveys the research focus, but it could be more specific regarding the ML techniques applied. A refined title such as “Machine Learning-Based Clustering and Yield Prediction in Ethiopian and Senegalese Sorghum Accessions Using Tree-Based Models” would improve clarity. The abstract effectively summarizes the study but could be more concise by focusing on the most impactful ML models rather than listing all tested algorithms. Additionally, performance metrics should be included, particularly R² and RMSE values, to quantify the effectiveness of the best-performing models. The author list and affiliations are well-structured, ensuring credibility.

A more refined title would make the research focus clearer, and reducing the number of ML models mentioned in the abstract would improve readability while keeping the key findings prominent. Including numerical performance metrics in the abstract would make the study’s contributions more tangible. Lastly, ending the abstract with a statement on real-world applications for sorghum breeding would reinforce its practical significance.

**A: We thank the reviewer for their suggestions to improve the clarity and impact of our manuscript. We have revised the title to accurately reflect our use of various machine learning methods. The abstract has been made more concise, focusing on the superior performance of tree-based models for yield prediction and including the corresponding R-squared and RMSE values.**

INTRODUCTION:

The introduction effectively contextualizes the study by discussing sorghum’s importance, its susceptibility to biotic and abiotic stresses, and the need for better classification and prediction techniques. The transition from traditional phenotypic methods to machine learning approaches is well-articulated. However, the section spends too much space discussing fungal diseases, which, while relevant, should not overshadow the main objective—the application of ML in sorghum classification and yield prediction. Additionally, basic ML definitions (supervised, unsupervised, and reinforcement learning) are unnecessary for an audience likely familiar with these concepts. The research gap is not explicitly stated, and it is unclear how this study differs from prior ML applications in plant breeding.

Reducing the discussion on fungal diseases would improve the focus on ML applications, and if necessary, these details could be moved to a separate background section. Removing basic ML definitions and instead elaborating on why clustering and predictive modeling are particularly useful for sorghum breeding would strengthen the introduction. Explicitly defining the research gap and explaining how this study advances previous ML applications in agriculture would make the introduction more compelling.

**A: We thank the reviewer for their valuable feedback on improving the clarity and focus of our Introduction. We have significantly reduced the discussion of fungal diseases, focusing on the broader context of biotic stresses. The unnecessary definitions of basic machine learning concepts have been removed. Most importantly, we have added a new paragraph explicitly stating the research gap our study addresses: the need for comparative evaluations of multiple machine learning methods for both clustering and yield prediction in diverse sorghum germplasm. Furthermore, we added clustering's usefulness.**

MATERIALS AND METHODS:

The materials and methods section is well-structured and provides details on data sources, statistical analysis, and machine learning models. The data description is clear, specifying the number of accessions and phenotypic traits analyzed. However, there is no mention of how categorical or non-numeric variables were handled, nor whether environmental variables such as soil quality or climate data were considered. The statistical analysis is robust, utilizing t-tests, PCA, and hierarchical clustering, but no justification is provided for selecting Ward’s linkage method. The ML analysis is comprehensive, covering feature scaling, train-test splitting, and hyperparameter tuning. However, the selection of ML models is not justified—why were Random Forest, XGBoost, and KNN chosen, and were other approaches like deep learning models (e.g., CNNs, LSTMs) considered? Additionally, clustering validity metrics (e.g., silhouette scores, Dunn index, Davies–Bouldin index) are not reported, making it difficult to assess the effectiveness of the clusters.

Clarifying how categorical or non-numeric variables were handled would make the methodology more transparent. Justifying the selection of Ward’s linkage for clustering would strengthen the statistical rigor of the study. Explaining why certain ML models were chosen over others and whether alternative ensemble or deep learning models were considered would enhance the credibility of the ML approach. Including clustering validity metrics would provide stronger validation for the identified clusters.

**We thank the reviewer for their thorough assessment of our Materials and Methods. All phenotypic traits, including disease scores, were treated as numerical values, and 'Cultivar' names were used only for labeling. While detailed environmental data were unavailable, we acknowledge this limitation. Ward's linkage was chosen for its tendency to produce compact clusters. Random Forest, XGBoost, and KNN were selected for their proven effectiveness in similar studies, and while deep learning was considered, it was deemed less suitable for our dataset size and structure. Finally, Silhouette scores have been added to assess clustering quality, as detailed in the revised manuscript.**

RESULTS:

The results section thoroughly presents findings from PCA, hierarchical clustering, and machine learning classification. PCA results successfully illustrate trait relationships and geographical clustering of sorghum accessions, reinforcing the role of genetic and environmental adaptation. However, the variance explained by the first two components (47.6%) is relatively low, suggesting that additional components may be needed for a more comprehensive interpretation. The hierarchical clustering analysis identifies four clusters, but the rationale for selecting four clusters is unclear—were silhouette scores or the elbow method used?

The machine learning classification results demonstrate that tree-based models significantly outperformed linear regression and SVMs, achieving accuracies above 70%. However, Table 2 lacks standard deviations or confidence intervals, making it difficult to assess result variability. The yield prediction analysis shows that the Boosted Tree model performed best (R² = 0.9026, RMSE = 1.9165), with seed weight and germination rate as the most influential traits. This aligns with prior studies but lacks a comparative discussion with other ML-based plant breeding studies. Additionally, potential overfitting in the Boosted Tree model is not addressed.

Justifying the selection of four clusters using silhouette scores or the elbow method would add statistical robustness to the clustering results. Reporting standard deviations/confidence intervals in Table 2 would improve the reliability of ML performance results. Discussing overfitting concerns in the Boosted Tree model and whether cross-validation was used to mitigate this issue would enhance the credibility of the model’s accuracy. Comparing findings with previous ML-based studies on crop yield prediction would place the study in a broader scientific context.

**A: We thank the reviewer for their detailed and helpful comments on our Results section. We agree that the initial PCA presentation focused primarily on the first two components; while these effectively illustrated key patterns, we acknowledge that further components capture additional variance. The choice of four clusters in the initial hierarchical clustering was based on visual inspection of the dendrogram, aimed at balancing cluster separation with a manageable number for subsequent machine learning analysis; now, we added silhouette scores for different machine learning methods. The original Table 2 has been replaced with a comprehensive evaluation using precision, recall, F1-score, and accuracy, including standard deviations, to address concerns about result variability. We also re-analyzed the Boosted Tree model with 5-fold cross-validation (10 repetitions) and validation set to mitigate overfitting, reporting revised performance metrics and the classification report for the test set. Finally, we have expanded the Discussion to include a comparison of our Boosted Tree model's performance with other relevant ML-based crop yield prediction studies, placing our findings within a broader scientific context.**

DISCUSSION:

The discussion effectively synthesizes key findings and links them to previous research. The geographical clustering of accessions is well-explained, and the link between rust resistance and East African climate conditions is insightful. The ML classification results are discussed thoroughly, emphasizing the advantages of tree-based models. However, there is no discussion on feature importance variability across models—were the same traits ranked important in different models? Additionally, the study does not explore whether the Boosted Tree model is generalizable to other sorghum populations.

The breeding implications are well-stated, particularly in emphasizing the importance of targeted trait selection. However, no mention is made of practical challenges in implementing ML in breeding programs. Furthermore, the role of environmental variables in yield prediction is ignored, despite their known impact on crop performance. The future research directions are promising, particularly the integration of genotypic data, but the potential for deep learning techniques (e.g., CNNs, transformers) is not discussed.

Discussing whether feature importance rankings were consistent across models would provide a more detailed understanding of trait significance. Addressing the generalizability of the Boosted Tree model to diverse sorghum populations would clarify its broader applicability. Mentioning practical challenges in applying ML to real-world breeding programs would make the discussion more applicable to agricultural research. Exploring the potential for integrating deep learning techniques into future research would align the study with cutting-edge advancements in ML.

**A: We thank the reviewer for their insightful comments on our Discussion. We have expanded the discussion of feature importance, comparing the top-ranked traits across the four best-performing models for yield prediction (Neural Boosted, Bootstrap Forest, SVM, and Boosted Tree) and presenting these comparisons in a new table. We also address the generalizability of the Neural Boosted model, acknowledging its limitations and the need for validation on diverse sorghum populations. Furthermore, we have added a discussion of the practical challenges of implementing machine learning in breeding programs, including data infrastructure, expertise, and cost-benefit considerations. As mentioned previously and in the revised manuscript, the lack of detailed environmental data is a limitation, and we have further emphasized its potential influence. Finally, we included that future studies could consider the use of deep learning methods with the availability of larger and more complex data set. These revisions enhance the comprehensiveness and practical relevance of our Discussion.**

CONCLUSION:

The conclusion effectively summarizes the study’s contributions and highlights the value of tree-based models in phenotypic trait analysis. It links findings to broader food security challenges, ensuring relevance. However, it does not mention whether these findings are generalizable to other crops. Additionally, a brief discussion on the broader applicability of ML approaches in plant breeding would enhance the paper’s impact.

Highlighting whether these ML methods can be applied to other cereal crops like maize and wheat would improve the generalizability of the findings. Discussing the potential integration of ML techniques in breeding pipelines would reinforce the study’s real-world impact.

**A: We thank the reviewer for their comments on the Conclusion. We have revised the Conclusion to explicitly address the potential generalizability of our findings to other cereal crops, such as maize and wheat. Furthermore, we have added a statement emphasizing the broader applicability of machine learning techniques for integrating into plant breeding pipelines. These additions strengthen the Conclusion by highlighting both the specific and general implications of our research.**

This study is well-structured and scientifically rigorous, presenting valuable insights into ML applications for sorghum breeding. However, improvements are needed in justifying ML model selection, reporting additional statistical metrics, addressing overfitting concerns, and discussing broader applicability. Addressing these points would enhance the study’s scientific impact and practical utility.

**A: We thank the reviewer for their overall positive assessment of our study and their constructive feedback. We appreciate the recognition of the study's structure, scientific rigor, and potential value for sorghum breeding.**
