# Supplementary material for: Seed quality drives grain yield in Ethiopian and Senegalese sorghum: Insights from machine learning
Source: PLoS One. 2025 Aug 14;20(8):e0329366. doi: 10.1371/journal.pone.0329366 (PMC12352784; doi:10.1371/journal.pone.0329366)
Supplement: Attachment — Submitted filename: Review.docx [file pone.0329366.s003.docx]

The manuscript presents an insightful study on how seed quality influences grain yield in Ethiopian and Senegalese sorghum using machine learning approaches. The study is well-structured and provides valuable contributions to agricultural research and food security. I am satisfied with all the comments. Now his paper is consider for further process.
