# Supplementary material for: Seed quality drives grain yield in Ethiopian and Senegalese sorghum: Insights from machine learning
Source: PLoS One. 2025 Aug 14;20(8):e0329366. doi: 10.1371/journal.pone.0329366 (PMC12352784; doi:10.1371/journal.pone.0329366)
Supplement: Attachment — Submitted filename: Reply to the reviwer R2.docx [file pone.0329366.s005.docx]

Dear Dr. Nguyen-Thanh Son and Reviewers,

Thank you for the opportunity to revise our manuscript. We appreciate the positive feedback and the constructive comments provided, particularly the detailed suggestions from Reviewer #4, which have helped us further strengthen the manuscript. Below, we provide a point-by-point response to the comments raised by Reviewer #4. Corresponding changes have been made in the manuscript, highlighted in yellow.

**Reviewer #4 Comments:**

**Comment 1: Clarity needed for the model labeled “NTanH(3)NBooste(8),” particularly regarding its architecture, neuron layers, and boosting method.**

**Response:** We thank the reviewer for highlighting the need for greater clarity regarding the specific Neural Boosted model architecture, correctly termed "NTanH(3)NBoost(8)". We have added the explanation to the materials and methods section.

**Comment 2: While ADASYN is appropriately used to handle class imbalance, a brief explanation of why it was preferred over alternatives like SMOTE would enhance methodological transparency.**

**Response:** We thank the reviewer for this suggestion. In the R1 manuscript, we introduced ADASYN in the Methods and compared classification results using undersampling versus ADASYN oversampling. In response to previous Reviewer #3 comments, we clarified that undersampling was used as a baseline, and ADASYN preserves the original data. More explanation was added in the materials and methods section.

**Comment 3: Clustering results yielded relatively low silhouette scores (0.32 and 0.31), indicating weak cluster separation. Acknowledging this and supplementing with dimensionality-reduction visualizations (PCA or t-SNE) would improve interpretability.**

**Response:** We thank the reviewers for their comments. As requested, we have now strengthened the wording in the discussion to more explicitly state that these modest scores indicate relatively weak separation between the clusters. While the reviewer suggests supplementing this with PCA or t-SNE visualizations related to the clustering results, we believe additional plots of this nature may not provide significant further insight beyond what is already conveyed by the modest silhouette scores and the existing PCA plot. Visualizing the ADASYN-oversampled data could also potentially be misleading due to the synthetic nature of the added data points. Therefore, we have focused on strengthening the textual interpretation of the silhouette scores in the discussion.

**Comment 4: Although the best model’s R² value of 0.36 is modest, it remains valuable given the absence of environmental or genotypic data. Briefly contextualizing this limitation would benefit the reader.**

**Response:** We thank the reviewer for this suggestion. We added in discussion that while the R-squared value of 0.36 is modest, it represents a meaningful prediction level achieved using only pre-harvest phenotypic traits, highlighting the challenge and value of phenotype-only prediction in the absence of environmental or genotypic data.

**Comment 5: The Materials and Methods section is rigorous ... restructuring it under subheadings like “Data Preprocessing,” “Feature Engineering,” and “Modeling Approaches” would improve clarity.**

**Response:** We appreciate the reviewer's suggestion to improve the structure and clarity of the Materials and Methods section using subheadings. To enhance clarity and better align with standard terminology for the initial data handling steps, we have renamed the first subheading from "Data source and characteristics" to "Data Preprocessing".

**Comment 6: A short rationale for selecting specific models (e.g., MLP, KNN, SVM) and justifying the choice of three clusters in K-means would strengthen the narrative. Mentioning whether clustering validation methods beyond silhouette score... were applied would add depth.**

**Response:** We thank the reviewer for suggesting we provide more rationale for our methodological choices regarding model selection and clustering validation.

**Comment 7: More detail on JMP Pro 17’s model selection process would also support reproducibility. Specifying which models were screened and on what basis they were selected... would be helpful.**

**Response:** We agree that more detail on the JMP Pro 17 process would improve reproducibility. The R1 Methods section mentions using the "Model Screening" function with K=5 fold cross-validation (10 repeats). The Results list the models included in Table 4, which were output by this screening.

**Comment 8: Inclusion of ADASYN improved classification metrics, and further discussion on the biological interpretation of trait clusters would be beneficial. Elaborating how trait clusters relate to known agronomic or adaptive differences... would enhance the biological insight.**

**Response:** We thank the reviewer for suggesting we enhance the biological interpretation. We elaborated the biological interpretation in the discussion as suggested.

**Comment 9: Explaining key metrics, like R² and RASE would aid accessibility.**

**Response:** We thank the reviewers for their comments. We have added brief explanations for R-squared and RASE.

**Comment 10: Noting sample size or environmental heterogeneity as potential limitations would round out the discussion.**

**Response:** We thank the reviewer for this suggestion. To clarify these points, we have explicitly added "the relatively small sample size (179 accessions)" in the discussion section.

**Comment 11: Suggestions for incorporating genomic or environmental variables in future work reflect a forward-looking approach. Emphasizing that integrating multi-modal data could significantly enhance model accuracy and biological relevance would strengthen this section.**

**Response:** We thank the reviewer for this suggestion. We have strengthened the concluding sentences of the discussion section's "Future research" paragraph and the final sentence of the conclusion.

**Comment 12: Minor formatting and typographical corrections are recommended to enhance clarity and polish.**

**Response:** We thank the reviewer for this reminder. We have thoroughly proofread the entire manuscript again to correct any remaining typographical errors and ensure consistent formatting.
